# Novel findings from 2,838 Adult Brains on Sex Differences in Gray Matter Brain Volume

**DOI:** 10.1038/s41598-018-38239-2

**Published:** 2019-02-08

**Authors:** Martin Lotze, Martin Domin, Florian H. Gerlach, Christian Gaser, Eileen Lueders, Carsten O. Schmidt, Nicola Neumann

**Affiliations:** 1 Functional Imaging Unit, Department of Radiology, University Medicine Greifswald, Greifswald, Germany; 2 Department of Psychiatry, University of Jena, Jena, Germany; 3 School of Psychology, University of Auckland, Auckland, New Zealand; 4 Laboratory of Neuro Imaging, School of Medicine, University of Southern California, Los Angeles, USA; 5 SHIP, Institute for Community Medicine, University Medicine of Greifswald, Greifswald, Germany

## Abstract

There is still disagreement among studies with respect to the magnitude, location, and direction of sex differences of local gray matter volume (GMV) in the human brain. Here, we applied a state-of-the-art technique examining GMV in a well-powered sample (n = 2,838) validating effects in two independent general-population cohorts, age range 21–90 years, measured using the same MRI scanner. More GMV in women than in men was prominent in medial and lateral prefrontal areas, the superior temporal sulcus, the posterior insula, and orbitofrontal cortex. In contrast, more GMV in men than in women was detected in subcortical temporal structures, such as the amygdala, hippocampus, temporal pole, fusiform gyrus, visual primary cortex, and motor areas (premotor cortex, putamen, anterior cerebellum). The findings in this large-scale study may clarify previous inconsistencies and contribute to the understanding of sex-specific differences in cognition and behavior.

## Introduction

Many brain structures present themselves as similar in men and women, especially when properly accounting for total brain size. It has therefore been proposed that both, brain structure and behavior, is largely overlapping between sexes^1^. However, one cannot assume a total absence of sex differences with regard to brain features as evidenced by both single studies and meta-analyses. In a gaussian-process regression coordinate-based meta-analysis including 16 voxel-based morphometry (VBM) studies, altogether comprising of 2,186 brain scans, Ruigrok and colleagues^2^ reported larger gray matter volumes (GMV) in women within the frontal pole, inferior/middle frontal gyrus, planum temporale/parietal operculum, anterior cingulate gyrus, right insular cortex, Heschl’s gyrus, thalamus, precuneus, parahippocampal gyrus, and lateral occipital cortex. In men, GMV was larger for the amygdala, hippocampus, parahippocampal gyrus, precuneus, putamen and temporal poles, the cingulate gyrus, as well as cerebellum^2^.

While meta-analyses have an enormous advantage over single studies in terms of statistical power, they are not immune to other pitfalls^3,4^, such as related to data acquisition, image analysis, and the (often manual) transfer of peak coordinates. Therefore, a study comparable in scale to meta-analyses, but not weakened by the typical methodological confounds would be desirable. On a similar note, studies investigating the reproducibility of sex effects in two independent cohorts using identical measurements and evaluation methods seem imperative.

Recently, in an UK biobank study on 5216 participants Ritchie and colleagues^5^ presented data on sex differences in total brain volume (preselected subcortical structures), cortical thickness (cortical structures), white matter, resting state connectivity and cognitive testing. Although this is the first study on such a large dataset, including different characteristic measurements of brain structure, connectivity and cognition, the investigated sample was not representative (mean age 62, range 44–77) years, higher education over-represented). Roughly, Ritchie and colleagues described larger brain volume in all preselected subcortical areas (except n. accumbens) of both hemispheres in men and increased cortical thickness in women. Men showed larger variance of brain volume measures than women.

However, an investigation of sex differences in the brain of a large representative cohort with (1) a broad age range, (2) correction for the total brain volume (TBV), (3) inclusion of a number of confounds known to affect brain volume, (4) using voxel-based morphometry for differentiating subcortical subregions is still lacking.

For the general differences between sexes, such as larger TBV, GMV and WMV in men^6^ and local cortical differences between men and women in the fronto-parietal (women > men) and occipito-temporal cortex (men > women), there seems to be increasing support^1,2,7^. In contrast, for subcortical structures, such as the hippocampus, inconsistent results have been reported. This inconsistency might result from differences in methodological approaches (lack of correction for TBV, global structure volume assessment in comparison to regional volume changes in VBM), but also from differences in cohort selection (age, sample size). For instance Neufang *et al*.^8^ found that testosterone levels predicted hippocampal size in younger females having larger hippocampi. Whereas sex differences in puberty and early adulthood may be particularly modulated by hormonal factors, in older adulthood environmental factors may have a greater impact. Therefore differences in younger collectives might well be absent in older cohorts, and vice versa. Large cohorts with a broad age range (21–90 years) like the current one may have the power to detect sex differences in small structures, such as the hippocampus, as a function of age.

Here, we compared male and female brains with respect to local (voxel-wise) gray matter volume in a large representative sample. First, we tested for reproducibility of effects in two independent cohorts (n1 = 967; n2 = 1,871). Since both showed highly reproducible results, the methods of collecting data were identical and the cohorts were not overlapping, we were able to combine both to perform one unified analysis. For this purpose, we applied a state-of-the-art brain mapping approach^9^ and analyzed 2,838 T1-weighted scans obtained from these two general population cohorts^10^. In addition, we investigated the interaction of sex and age for the GMV in the hippocampus, since previous findings with regard to this structure were highly controversial and an impact of age on the hippocampal GMV can be assumed.

## Results

### Cohort 1 (n = 967) and Cohort 2 (n = 1,871)

Analyzing Cohort 1, in women (women > men), on average larger GMV was prominent in bilateral prefrontal areas, such as the ventrolateral prefrontal cortex (vlPFC, BA 47), the medial and lateral orbitofrontal cortex (OFC), the anterior cingulate cortex, the frontal pole, and the dorsolateral prefrontal cortex (dlPFC, namely BA 45, 46). In addition, women on average showed larger GMV in the right gyrus of Heschl, the bilateral lateral occipital lobe, posterior insula, the right superior parietal lobe (SPL), the bilateral superior temporal sulcus (STS), and the left posterior cerebellar hemisphere. Effect sizes (Cohen’s d) for women > men ranged from 0.30 (frontal pole) to 0.45 (lateral OFC). Findings are further detailed in Supplementary Table 1A. In men (men > women), on average larger GMV was evident in bilateral temporal areas, such as the parahippocampal gyrus, the hippocampus (Hi), the amygdala (Am), the temporal pole (TP), and the fusiform gyrus (FG), as well as the bilateral putamen (Pu), anterior cerebellar (aCBH, Larsell’s lobule IV-VII), and left primary visual cortex (BA 17, 18). Effect sizes (Cohen’s d) for men > women ranged from 0.27 (vlPFC) to 0.49 (parahippocampal gyrus). Findings are further detailed in Supplementary Table 1B.

Analyzing Cohort 2, reproduced all of the aforementioned effects. That is, all areas reported for Cohort 1 were also evident for Cohort 2 (see Supplemental Tables 1 and 2). We therefore repeated the analyses pooling Cohort 1 and Cohort 2 (see next section).

### Combined Cohort (n = 2,828)

**Figure 1:**
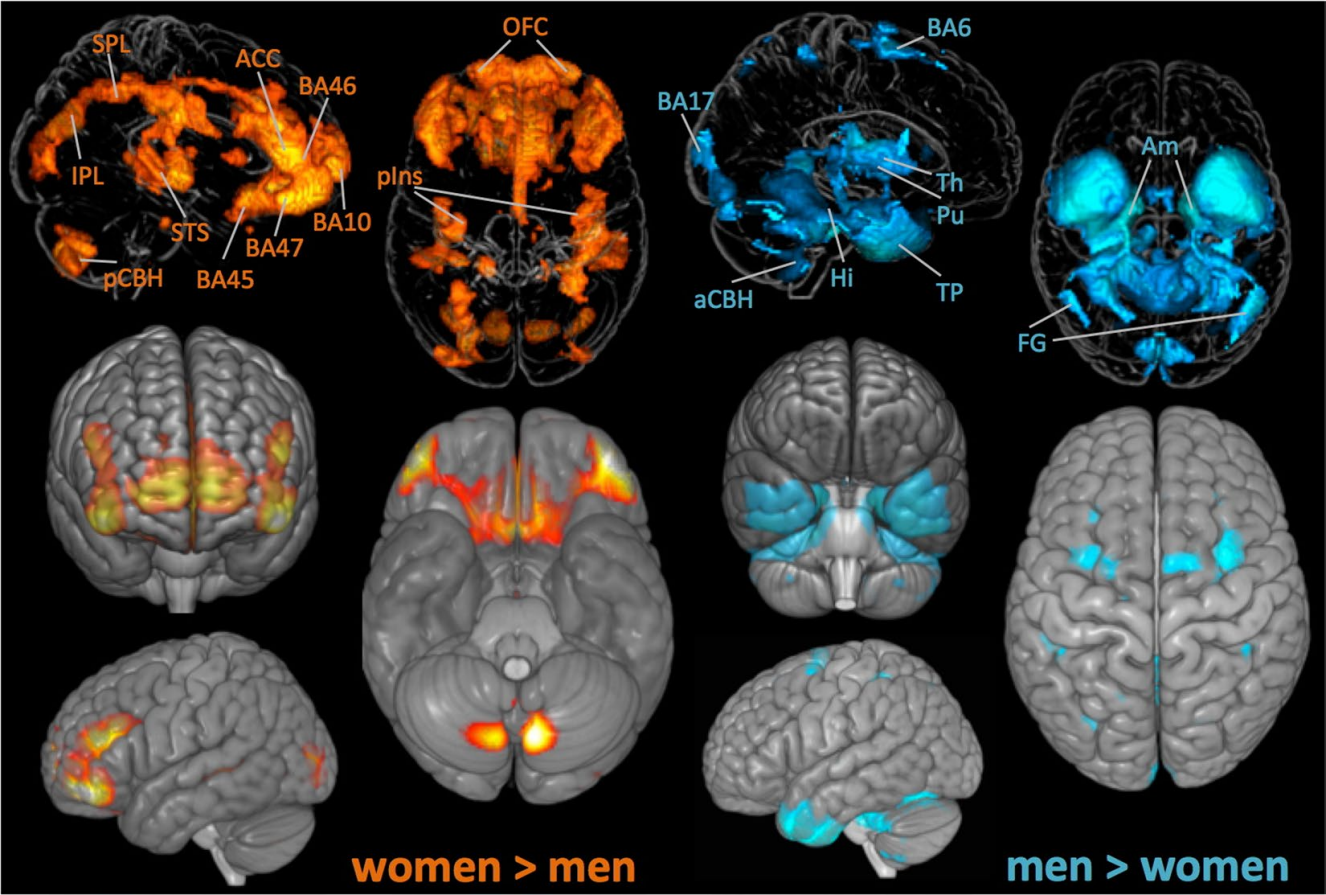
Significant sex differences for the combined cohort (n = 2,838). Glass brain projections with labels (top) and MNI-standard brain projections (bottom). Orange clusters display regions with larger gray matter volume in women (women > men): pCBH = posterior cerebellar hemisphere; IPL = inferior parietal lobe; SPL = superior parietal lobe; STS = superior temporal sulcus; ACC = anterior cingulate cortex; BA = Brodmann areas 45, 46, 47, 10; OFC = orbitofrontal cortex; pIns = posterior insula. Blue clusters display regions with significantly larger gray matter volume in men (men > women): BA = Brodmann areas 6, 17; aCBH = anterior cerebellar hemisphere, Hi = hippocampus, Th = thalamus, Pu = putamen, TP = temporal pole, FG = fusiform gyrus, Am = amygdala). All findings are significant at p ≤ 0.05, FWE corrected for multiple comparisons.

Age did not differ between men and women. Men indicated longer education (12.97 ± 2.50 years) than women (12.44 ± 2.31 years; p < 0.001). As shown in Fig. 1, on average, women had larger GMV (women > men) in bilateral vlPFC (BA47), medial and lateral OFC, ACC, frontal pole (BA 10), lateral occipital lobe (BA 19), right Heschl gyrus, bilateral dlPFC (BA 45,46), posterior insula, precuneus, STS, left thalamus and SPL and right posterior cerebellar hemisphere and IPL. On average, men had larger GMV (men > women) in bilateral parahippocampal gyrus and hippocampus, amygdala, temporal pole, putamen, fusiform gyrus, anterior cerebellar hemisphere, primary visual cortex (BA 17), and premotor cortex (BA 6).

When testing an interaction of age (median (53 years) split of the sample) and sex we found a significant effect for the hippocampus. Post hoc t-tests demonstrated that older women (≥53 years) had larger posterior-superior hippocampal GMV (t = 5.52; Cohens d = 0.21; 141 voxels in ROI; MNI-coordinates: −36, −36, −9) than older men. In contrast, younger men had larger GMV in the anterior-inferior hippocampus than younger women (t = 8.21; Cohens d = 0.31; 603 voxels in ROI; MNI-coordinates: −21, −2, −22). Both effects were only observed for the left hemisphere. The comparisons older men > older women and younger women > younger men revealed no significant effects.

Effect sizes for women > men were as large as d = 0.38 (prefrontal cortex) and for men > women as large as d = 0.53 (parahippocampus). A detailed list of different regions between men and women is provided in Tables 1 and 2.

**Table 1:** Whole Sample (n = 2,838): *Women* > *Men*.

Region	Hemisphere	t-value	Cohens d	Cluster size (voxel)	MNI coordinates		
					x	y	z
Ventrolateral prefrontal cortex (BA47)	L R	10.05 8.38	0.38 0.32	169 70	−48 50	44 44	−15 −15
Medial orbitofrontal cortex (OFC)	L R	9.31 8.14	0.35 0.31	3593	−15 9	24 26	−16 −16
Anterior cingulate cortex (ACC)	R L	9.22 8.05	0.35 0.30	3087	10 −8	46 44	−2 14
Frontal pole (BA10; FP1&2))	L R	9.15 8.71	0.34 0.33	5452	−10 10	69 70	8 3
Lateral occipital lobe (BA19)	L R	8.77 6.29	0.33 0.24	2229 756	−26 38	−82 −88	20 8
Heschl gyrus (A1)	R L	8.72 5.89	0.33 0.22	89 11	42 −39	−21 −24	0 0
Dorsolateral prefrontal cortex (BA45)	L R	8.91 7.85	0.34 0.30	193 123	−46 50	30 32	22 22
Dorsolateral prefrontal cortex (BA46)	L R	7.11 7.30	0.27 0.27	227 396	−33 34	40 42	4 4
Posterior insula (pIns)	L R	6.48 8.31	0.24 0.31	199 349	−42 40	−15 −16	−2 0
Inferior parietal lobe (IPL)	R L	7.00 6.03	0.26 0.23	122 47	44 −42	−24 −34	36 36
Precuneus	L R	6.57 5.07	0.25 0.19	163 59	−15 15	−42 −40	45 44
Superior parietal lobe (SPL)	R L	7.50 6.57	0.28 0.25	101 182	39 −15	−34 −42	38 45
Superior temporal sulcus (STS)	L R	7.26 6.91	0.27 0.26	680 658	−45 48	−34 −30	0 4
Posterior cerebellum (pCB)	L R	9.75 6.63	0.37 0.25	371 86	−9 18	−72 −75	−50 −52
Thalamus	L	5.50	0.21	79	−3	−10	9

**Table 2:** Whole Sample (n = 2,838): *Men* > *Women*.

Region	Hemisphere	t- value	Cohen’s d	Cluster size	MNI coordinates		
					x	y	z
Parahippocampal gyrus	L R	13.82 13.57	0.53 0.52	933 849	−18 20	−8 −10	−30 −30
Hippocampus (Hi)	L R	12.50 11.82	0.47 0.44	133 89	−18 16	−12 −10	−27 −26
Amygdala (Am)	L R	12.20 9.80	0.46 0.37	402 258	−18 21	−6 −2	−27 −28
Thalamus (Th)	L R	10.01 8.62	0.38 0.32	101 174	−22 22	−12 −12	8 8
Temporal pole (TP)	L R	9.88 7.72	0.37 0.29	1173 632	−34 21	14 3	−44 −38
Putamen (Pu)	L R	8.90 8.14	0.33 0.30	302 523	−24 26	0 −2	8 4
Fusiform gyrus (FG)	L R	9.88 9.30	0.37 0.35	636 1212	−34 34	14 −18	−44 −36
Anterior cerebellar hemisphere (aCBH)	R L	9.00 8.30	0.34 0.31	1515 1531	33 −16	−30 −46	−32 −18
Occipital areas (BA17)	L R	6.52 5.83	0.24 0.22	75 10	−14 12	−54 −86	−8 −15
Premotor Cortex (BA6)	L R	6.15 7.96	0.30 0.23	325 496	−30 32	0 0	66 66

## Discussion

The current study compared sex differences in the brain examining gray matter volume in two independent cohorts. We found a high reproducibility of effects between cohorts and therefore pooled the data for a unified analysis, which resulted in a well-powered sample (n = 2,838). Since this study did not directly measure associations between brain structure and behavior interpretations drawn between brain structure and behavioral implications are speculative.

### Correspondence with previous findings

In our study, the most compelling differences between cortical GMV of men and women laid in the larger prefrontal GMV in women and larger anterior-medial temporal GMV in men. This confirms results of Chen and colleagues^7^ describing regional GMV differences in an cohort of 411 middle-aged healthy participants (44–48 years) with men > women in midbrain, left inferior temporal gyrus, right occipital lingual gyrus, right middle temporal gyrus, and both cerebellar hemispheres and women > men in dorsal anterior, posterior and ventral cingulate cortices, and right inferior parietal lobule. In addition, the present study largely confirmed the meta-analytic findings by Ruigrok and colleagues^2^. That is, we detected larger GMV in women in the inferior and middle frontal gyrus, the ACC, the right OFC, the right insula, the lateral occipital cortex, the Heschl gyrus, the thalamus, the precuneus, but not in the planum temporale/Wernicke’s area.

### GMV-differences in subcortical structures (parahippocampus, hippocampus, thalamus)

For the parahippocampus, Ruigrok and colleagues^2^ reported larger GMV posteriorly in women, and larger GMV anteriorly in men. Interestingly, the parahippocampus showed the strongest sex effect (men > women) in the present study and we did not observe any effect for women > men in this area. For the parahippocampal gyrus, Ritchie and colleagues^5^ reported that females showed relatively higher thickness but males showed relatively higher volume and surface area.

In the current study, the GMV in the anterior-inferior hippocampus was larger in men than in women. However, testing the interaction of age and sex, this held true only for the younger part of the sample (median split, (<53 years), but not for the older (≥53 years). In contrast, older women showed increased left posterior-superior hippocampal GMV compared to older men. It might well be the case that for women hormonal changes after menopause modulate these specific hippocampal GMV differences in comparison to men^11^. Additional information on this effect is provided in the Supplement. In accordance with our study (but measured for the complete structure volume), Ritchie and colleagues^5^ (mean age 62 years) reported no sex differences in hippocampal volume after correction for total brain volume. Our results are also corroborated by the meta-analysis of Ruigrok *et al*.^2^, showing increased hippocampal volume bilaterally for men.

We found larger GMV of the thalamus in men compared to women in contrast to Ruigrok and colleagues^2^ (increased thalamic GMV in females), except for the left thalamus, where we found a larger GMV for the posterior part in women. This demonstrates the strength of a voxelwise analysis enabling a more detailed analysis of subregions.

### Larger GMV in men in motor areas

For men compared to women, we observed larger GMV in the putamen, the premotor cortex (BA6), and the anterior cerebellum (i.e., structures involved in motor function). Ruigrok *et al*.^2^ likewise found larger GMV in men in the bilateral putamen, bilateral cerebellum and the left precentral gyrus. Larger GMV in motor areas in men may arise during the phases when testosterone in boys and estradiol in girls are causing the greatest modulation of the brain^8^.

### Larger GMV in women in prefrontal areas

Increased GMV in women’s prefrontal areas has been reported in a number of smaller studies and was therefore the most prominent result in the large meta-analysis by Ruigrok *et al*.^2^. The present study confirms these results with women demonstrating larger GMV in bilateral dorso- and ventrolateral prefrontal cortices, the frontal pole, and the medial orbitofrontal cortex. In contrast to Ritchie *et al*.^5^, who were speculating about the functional meaning of higher prefrontal GMV in men as “regions that showed the largest effects were broadly areas involved in the hypothesized intelligence-related circuit in the “P-FIT” model“, we demonstrated the contrary with females showing larger GMV in the same areas. Although our study did not measure cognitive or behavioral data, and is thus not able to draw conclusions about cognitive functioning and brain structure, we would like to point out that increased GMV is usually associated with a better functioning in the cognitive domain^12^. Prefrontal areas with larger GMV in women are functionally important for executive functioning^13^, such as planning, working memory, inhibition, mental flexibility as well as the initiation and monitoring of action, but also for emotional control, moral considerations^14^ and processing of language^15^.

### Do differences between men and women do not allow for individual assignment?

Although these sex differences have been robustly observed in different cohorts, a relevance for an individual is rather small: Joel and colleagues demonstrated that there is a considerable overlap between the features of brain form between males and females and that these features are internally inconsistent^1^, even when considering only those showing the largest sex differences. In response to the Joel *et al*.^1^ study, Chekroud *et al*.^16^ used a multivoxel pattern analysis to distinguish male and female brains by structural differences. They found a classification accuracy of 93–95% and concluded that sex can be reliably predicted by brain structure when considering the brain mosaic as a whole.

### Limitations

Brain structural differences between men and women are the result of complex biological and environmental influences and the underlying neural mechanisms a matter of ongoing discussion. Additionally, no complete understanding exists whether more GMV is associated with improved function, even if most studies comparing experts and non-experts or longitudinal studies applying training paradigms demonstrated specifically increased GMV in those areas functionally representing improved performance^17–19^. However, these associations are poorly understood and a matter of ongoing discussions^20^.

Furthermore, while cognitive function is associated with GMV, it has also been linked to white matter and structural connectivity between different brain regions^21^. Thus, gray matter may explain some, but not all of the differences. In addition, sex-specific incidence of pathologies may have an impact on differences in GMV between men and women. In the current study, all pathologic brain scans had been excluded in this sample, as described in the Methods.

Finally, different measuring techniques of GMV do only partially provide comparable results. A major drawback of voxel-based measurements is that they combine cortical thickness and surface area into one single measurement. It has been demonstrated that vertex-based measures (cortical thickness, surface area) are more or less independent of each other^22^. A global or local change of these measures in different directions (e.g. increase of cortical thickness, decrease of surface area) wouldn’t necessarily be visible in voxel-based morphometry, and this may be one principal explanation for the differences between vertex- and voxel-based measures.

## Conclusion

The outcomes of this large-scale study offer an excellent starting point for follow-up research elucidating the role of a sex-specific brain anatomy for cognitive, emotional, and behavioral differences between men and women. In particular, the combination of brain morphology and behavioral testing of cohorts is a challenge for the future. Moreover, they may help to explain sex differences in the prevalence and progression for a number of disorders, diseases, and disabilities.

## Methods and Materials

### Sample and Imaging

The Study of health in Pomerania (SHIP) comprises two independent general population cohorts, SHIP and SHIP-TREND. The primary objectives of SHIP were (i) to assess prevalence and incidence of common risk factors, subclinical disorders and clinical diseases; and (ii) to investigate the complex associations between the aforementioned issues.

Participants were selected from West Pomerania in Northeastern Germany. Inclusion criteria were primary place of residence in the target area and age 20–79 at sampling. No other criteria were employed for exclusion or inclusion to obtain a general population sample as representative as possible. Invitations comprised three written invitations, phone calls, and personal contacts.

In total, out of 6,265 eligible individuals, 4,308 participated (response 68.8%) in the SHIP-0 baseline examinations (1997–2001). Follow-ups took place from 2002–2006 (SHIP-1, N = 3300) and from 2008–2012 (SHIP-2, N = 2333). SHIP-Trend was a new cohort established in 2008. Out of 8826 eligible subjects 4420 (2,275 women) participated (response 50.1%). Both cohorts showed no overlap since a selection criteria of SHIP-TREND was no participation in SHIP-0, a baseline examination of SHIP-2. In total 3371 out of 6753 SHIP-2 and SHIP-Trend participants took part in the MRI examination. High-resolution magnetic resonance imaging (MRI) data for this project were available from n = 1,182 SHIP-2 and from n = 2,186 SHIP-Trend-0 participants. For further details of the procedures involved in the selection of participants and amount of data gathered please refer to^10,23^. Table 3 is providing the descriptive data for the entire sample. The age range was 21–90 years.

**Table 3:** Demographic data for the two cohorts.

Data	TBV [ccm]	IQR	Age [years]	Edu [years]	Nicotine [packyears]	Alcohol [ml/30d]	BMI
Men (SD)	1644 (123)	2.84 (0.34)	52.30 (14.14)	12.97 (2.50)	10.86 (16.34)	13.83 (15.71)	28.12 (3.73)
Women (SD)	1448 (105)	2.67 (0.28)	52.43 (13.19)	12.44 (2.31)	4.61 (9.64)	4.52 (6.23)	27.19 (4.96)
Difference p-value	0.001	0.001	0.79	0.001	0.001	0.001	0.001

Abbreviations: TBV: total brain volume; IQR: index of quality rating; Edu: years of education; BMI: body mass index.

The study protocol was approved by the Ethics Committee of the University Medicine of Greifswald and written informed consent was obtained from each subject. In addition, all methods were performed in accordance with the relevant guidelines and regulations. All brain images were obtained on the same 1.5 Tesla Siemens MRI scanner (Magnetom Avanto, Siemens Medical Systems, Erlangen, Germany) without software updates during the evaluation period. More specifically a T1-weighted magnetization prepared rapid acquisition gradient echo (MPRAGE) sequence was used with the following parameters: 176 slices, matrix = 256 × 176 pixels, voxel size = 1.0 mm isotropic, slice thickness = 1.0 mm, repetition time = 1900 ms, echo time = 3.37 ms, flip angle 15°.

### Quality control and exclusion of pathologies

All MRI head scans were visually inspected with regard to image artifacts and clinical abnormalities. Any brain images indicating stroke, multiple sclerosis, epilepsy, Parkinson’s disease, dementia, cerebral tumor, intracranial cyst or hydrocephalus were excluded, leaving 1,081 (SHIP-2) and 2,046 (SHIP-Trend-0) images. Furthermore, subjects with recorded intake of anxiolytics or opioids, as well as with PHQ9 (Patient Health Questionnaire with 9 responses) depression scores^24^ greater than 14 were excluded, leaving 1,037 (SHIP-2) and 1,984 (SHIP-Trend-0) images. Finally, all subjects with incomplete datasets for possible confounds (i.e., age, years of education, nicotine intake, alcohol consumption, body mass index) were excluded. The final sample contained 2,838 subjects, with 967 subjects from SHIP-2 and 1,871 subjects from SHIP-Trend-0. We differentiated “sex” as the item “man“ or “women” as provided by verbal questionnaire by the participant.

### Data preprocessing

T1-weighted images were preprocessed in MATLAB (The MathWorks, Natick, MA) using Statistical Parametric Mapping, version 12 (SPM12; Wellcome Department of Cognitive Neurology, University of London) and the Computation Anatomy Toolbox (CAT) for SPM (CAT 12; Christian Gaser; Department of Psychiatry, University of Jena) with CAT12 default parameters, as described elsewhere^25^. Briefly, images were corrected for magnetic field inhomogenities, spatially normalized using the DARTEL algorithm^26^, and segmented into GM, white matter (WM), and cerebrospinal fluid (CSF). The segmentation process was further enhanced by accounting for partial volume effects^27^ and by using a hidden Markov Random Field (MRF) model^28^. Finally, the resulting GM segments were smoothed using a Gaussian kernel of 8 mm full width at half maximum (FWHM). In addition, all scans underwent an automated quality check, revealing an index of quality rating (IQR), which later was used as an additional covariate in the statistical model. Total brain volume (TBV) was calculated as sum of GM, WM, and CSF, (also to be used later as a statistical covariate).

### Statistical analyses

We first investigated whether there were significant GMV differences between men and women in cohort 1 (SHIP-2; n = 967) and cohort 2 (SHIP-Trend-0; n = 1,871), separately. Then, we tested for significant differences between those cohort-specific effects (SPM, two sample t-test). Since the absence of a significant effect between two cohorts does actually not allow to consider that both groups are equivalent we used a modified strategy as suggestions by Lakens^29^ for our voxel based statistical approach. The highest trend for an effect between cohorts was observed for the contrast SHIP2 minus Trend0 in the right BA47 with a t-value of 3.80. When calculating an effect size for this t-value (considering group sizes, two sample t-test for independent means of the two groups) we found an effect size (g*power, version 3.1) of Cohens d = 0.29 not relevant according to Lakens^29^. After ensuring that there were no GMV sex differences between the cohorts, we finally evaluated both cohorts together (combined; n = 2,838). For this purpose, a full factorial model as implemented in SPM12 was applied, while removing the variance associated with the following variables: TBV, IQR, age, years of education, nicotine intake, alcohol consumption, and body mass index (BMI). Alpha was set at p < 0.05, and corrections for multiple comparisons were applied using the family-wise error (FWE) rate. Clusters smaller than 10 voxel were not considered.

### Anatomical labeling

The anatomical differentiation of significant effects was predominantly performed with ANATOMY, version 2.2b^30^. For regions that have not yet been classified cytoarchitecturally using ANATOMY, the most appropriate differentiations suggested by other atlases were applied. That is, for BA 46 we used Sallet *et al*.^31^, for the insula we used Neuromorphometrics (Neuromorphometrics, Inc.) as provided with the SPM12 package, and for the cerebellum, putamen, temporal pole and fusiform gyrus, we used the AAL atlas^32^.

## Supplementary information

Supplementary
