# Supplementary material for: Novel findings from 2,838 Adult Brains on Sex Differences in Gray Matter Brain Volume
Source: Sci Rep. 2019 Feb 8;9:1671. doi: 10.1038/s41598-018-38239-2 (PMC6368548; doi:10.1038/s41598-018-38239-2)
Supplement: Supplementary file 1 — Supplementary [file 41598_2018_38239_MOESM1_ESM.pdf]

Title of the manuscript: "Novel findings from 2,838 Adult Brains on Sex Differences in Gray Matter Brain Volume"

Authors: Martin Lotze, Martin Domin, Florian H. Gerlach, Christian Gaser, Eileen Lueders, Carsten Schmidt, Nicola Neumann

Supplementary Table 1A. Cohort 1 – SHIP2 (n=936): *Women > Men*

**Region**	**Hemisphere**	**t- value**	**Cohens d**	**Cluster size**	**MNI coordinates**		
					**x**	**y**	**z**
Ventrolateral prefrontal	L	6.11	0.40	39	-45	40	-14
cortex (BA47)	R	5.01	0.32	7	46	42	-15
Medial OFC	L	5.55	0.36	189	-18	24	-16
	R	5.22	0.34	46	20	26	-15
Lateral OFC (BA47)	L	6.94	0.45	65	-48	44	-15
	R	5.14	0.33	35	22	14	-24
ACC	R	6.37	0.41	134	12	46	-3
Frontal pole (BA10)	L	4.69	0.30	16	-10	51	-8
	R	5.75	0.37	123	10	66	4
DLPFC (BA45)	L	5.23	0.34	21	-45	27	24
	R	4.79	0.31	13	50	30	22
DLPFC (BA46)	R	5.89	0.38	74	34	38	9
Lat. occipital lobe	L	5.45	0.36	38	-28	-82	20
Heschl gyrus	R	5.46	0.36	16	40	-18	3
Posterior Insula	L	4.80	0.31	15	-42	-15	-2

	R	5.57	0.36	85	42	-15	-2
SPL	R	4.61	0.30	1	12	-48	50
STS	R	4.84	0.31	54	48	-34	4
	L	4.98	0.32	39	-45	-34	-0
pCBH	L	6.59	0.43	183	-9	-72	-48

Supplementary Table 1b. Cohort 1 – SHIP2 (n=936): *Men > Women*

Region	Hemis-phere	t	Cohens D	Cluster size	MNI coordinates		
					x	y	z
Parahippocampal gyrus	L	6.6	0.43	456	-20	-8	-32
	R	7.6	0.49	321	20	-8	-33
Am	L	6.49	0.42	126	-21	-4	-32
	R	5.44	0.35	24	21	-2	-28
Hi	R	7.02	0.45	22	16	-10	-26
	L	7.02	0.45	24	-15	-12	-26
TP	L	6.41	0.41	325	-39	10	-45
	R	5.11	0.33	73	46	15	-42
Th	L	6.11	0.40	25	-22	-12	8
	R	5.34	0.35	21	22	-12	8
Pu	R	6.04	0.39	393	24	-8	9
	L	6.08	0.39	195	-24	-6	9
FG	L	6.23	0.40	322	-32	12	-46
	R	6.20	0.40	202	22	-8	-38
Occipital (BA17,18)	L	4.75	0.31	2	-14	-51	-6
aCBH	R	5.33	0.34	338	21	-30	-32
	L	5.76	0.37	549	-9	-45	-3

Supplementary Table 2a. Cohort 2 – SHIP-Trend-0 (n=1,902): *Women > Men*

**Region**	**Hemisphere**	**t- value**	**Cohens d**	**Cluster size**	**MNI coordinates**		
					**x**	**y**	**z**
Medial OFC	L	7.83	0.36	2355	-9	28	-18
	R	7.48	0.35	2355	6	24	-18
Lateral OFC	L	7.67	0.35	122	-50	44	-12
	R	7.36	0.34	71	50	44	-15
ACC	L	8.32	0.38	2732	-6	44	15
	R	6.97	0.32		10	46	-2
Frontal pole (BA10)	L	8.21	0.38	989	-8	64	3
	R	5.96	0.28	211	6	68	6
Ventrolateral prefrontal	L	5.84	0.27	16	-46	40	-6
cortex (BA47)	R	6.02	0.28	15	46	42	-12
Lateral occipital lobe	L	6.81	0.31	135	-38	-87	3
(BA19)	R	5.02	0.23	18	38	-86	6
Heschl gyrus	R	6.26	0.29	29	40	-18	3
DLPFC (BA45)	L	8.30	0.38	175	-48	33	21
	R	6.48	0.30	68	50	32	22
DLPFC (BA46)	L	5.69	0.26	213	-21	60	16
	R	6.01	0.27	278	38	44	0
pIns	L	5.10	0.24	26	-39	-20	-2

	R	6.72	0.31	229	39	-18	-2
IPL	R	5.70	0.26	208	58	-24	24
precuneus	L	4.83	0.22	9	-10	-40	46
	R	5.19	0.24	25	0	-68	45
SPL	L	5.65	0.26	147	-15	-44	46
	R	4.90	0.23	17	-2	-69	42
STS	L	5.32	0.25	326	-48	-38	6
	R	5.72	0.26	468	51	-36	9
pCBH	R	5.58	0.26	43	18	-75	-52
	L	7.59	0.35	162	-9	-72	-51
Thalamus	L	5.15	0.24	62	-3	-10	8

Supplementary Table 2b. Cohort 2 – SHIP-Trend-0 (n=1,902): *Men > Women*

Region	Hemisphere	t- value	Cohens d	Cluster size	MNI coordinates		
					x	y	z
Parahippocampal gyrus	L	11.97	0.55	807	-18	-8	-30
	R	11.37	0.53	812	20	-12	-30
Am	L	11.14	0.52	489	-18	-6	-27
	R	8.83	0.41	244	21	-2	-28
Hi	L	11.18	0.52	118	-18	-12	-27
	R	10.12	0.47	41	21	-10	-28
TP	L	8.97	0.41	1390	-34	14	-45
	R	6.77	0.31	494	21	3	-38
Th	L	8.52	0.39	93	-22	-12	8
	R	6.90	0.32	128	22	-12	8
Pu	L	9.02	0.42	462	-24	-6	10
	R	7.88	0.36	547	24	-10	10
FG	L	9.02	0.42	624	-34	15	-45
	R	8.44	0.39	839	22	-12	-36
Occipital areas (BA17,18)	R	7.64	0.35	840	-6	-86	-16
	L	4.90	0.23	22	-12	-84	-18
aCBH	R	7.87	0.36	6231	21	-30	-32
	L	7.17	0.33	6231	-28	-50	-34

Supplementary Figure 1

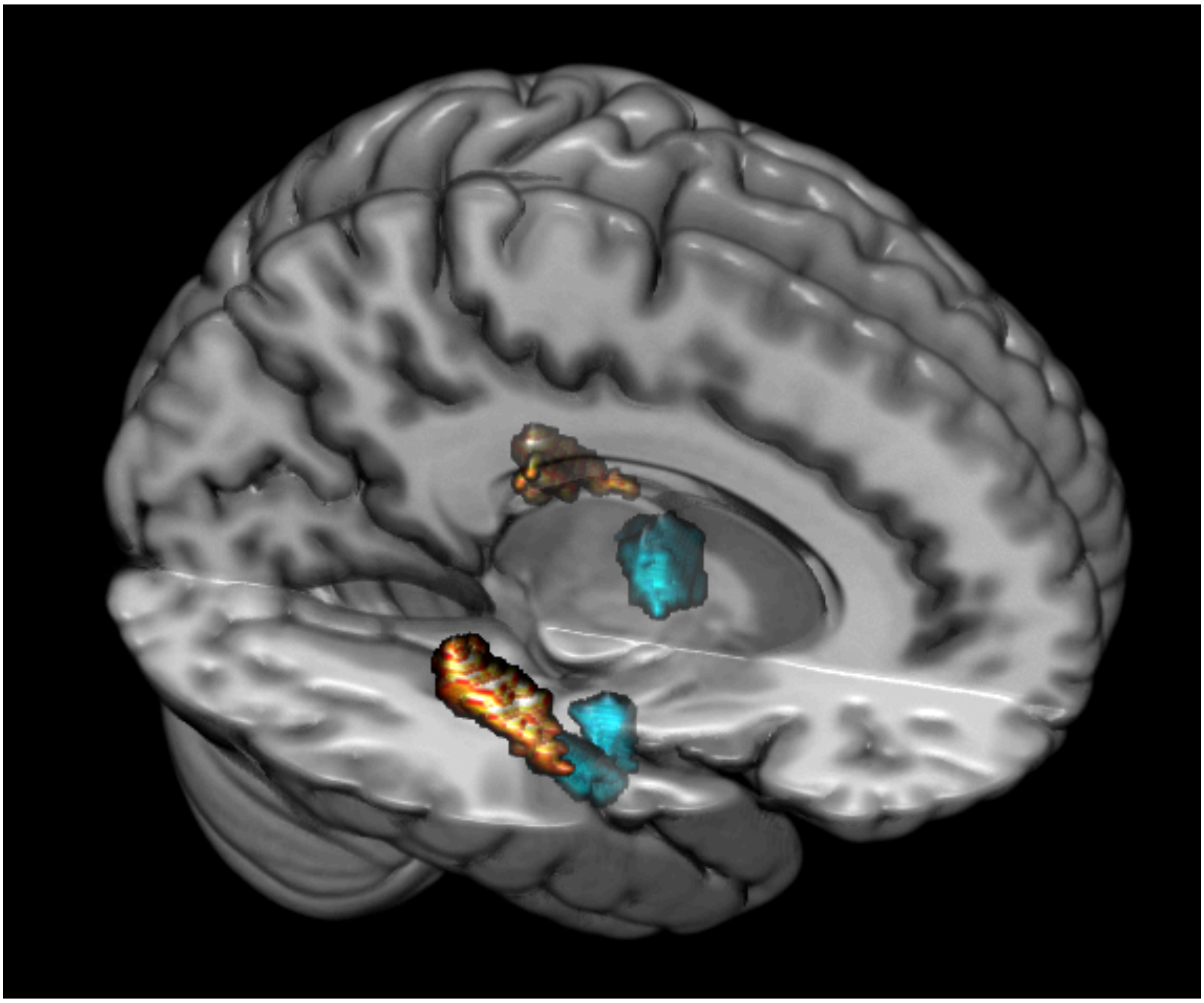

Legend Supplementary Figure 1:

The controversial results on the hippocampal volume in the previous literature between men and women were tested by calculating an interaction of sex and age. Therefore the sample was divided by a median split in an younger (<53 years) and an older sample (≥53 years). Younger men (cool blue) showed higher anterior hippocampal GMV in comparison to younger women (right hippocampus: t=6.03; cluster: 306; p<0.0001; coordinates: 38, -32, -10; left hippocampus: t=5.46; cluster: 171; p=0.001; coordinates: -34, -36, -19). In contrast older women (hot orange) showed higher posterior hippocampal GMV compared to older men (left hippocampus: t=12.96; cluster: 401; p<0.0001; coordinates: -18, -12, -27; right hippocampus: t=12.29; cluster: 203; p<0.0001; coordinates: 16, -10, -26). The statistical threshold was p<0.05, FWE corrected over the whole brain volume. We masked the resulting map for the hippocampus and overlaid on a segmented MNI-brain using MRIcroGL (FPS 10.1).
